# Impact of Examined Lymph Node Count on Precise Staging and Long-term Survival After Neoadjuvant Therapy for Carcinoma of the Esophagus: A SEER Database Analysis

**DOI:** 10.3389/fsurg.2022.864593

**Published:** 2022-04-29

**Authors:** Tao Bao, Lei Bao, Wei Guo

**Affiliations:** ^1^ Department of Thoracic Surgery, Daping Hospital, Army Medical University, Chongqing, China; ^2^ Computer Teaching and Research Office, Army Academy of Artillery and Air Defense, Hefei, China

**Keywords:** esophageal carcinoma, esophagectomy, neoadjuvant treatment, lymphadenectomy, survival, SEER

## Abstract

**Purpose:**

To identify the optimal number of lymph nodes dissected during esophagectomy following neoadjuvant therapy for carcinoma of the esophagus by using the Surveillance, Epidemiology and End Results Registry (SEER) database.

**Patients and Methods:**

Patients who underwent neoadjuvant Chemoradiotherapy (nCRT) plus esophagectomy with EC from 2001–2016 were analyzed retrospectively in the SEER database. We analyzed the correlation between the lymphadenectomy count and nodal stage migration and overall survival (OS) by using a binary logistic regression model and Cox proportional hazards regression. The curves of the odds ratios (ORs) of nodal stage migration and hazard ratios (HRs) of OS were smoothed using the LOWESS technique, and the cutoff points were determined by the Chow test. The OS curves were calculated with the Kaplan-Meier method.

**Results:**

Among the 4,710 patients analyzed in the SEER database, a median of 12 lymph nodes (IQR, 7–19) were harvested. There was a significantly proportional increase in nodal stage migration (OR, 1.017; 95% CI, 1.011 to 1.023; *P *< 0.001) and serial improvements in OS among node-negative patients (HR, 0.983; 95% CI, 0.977 to 0.988; *P *< 0.001) with an increased ELN count after adjusting for the T stage. The corresponding cutoff point of the 16 ELNs was calculated for the OR of stage migration by the Chow test. For those with node-negative and node-positive diseases, no significant trend of survival benefit that favored a more extensive lymphadenectomy was demonstrated (HR, 1.001; 95% CI, 0.989 to 1.012; *P *= 0.906; and HR, 0.996; 95% CI, 0.985 to 1.006; *P *= 0.405, respectively).

**Conclusion:**

On the basis of these results, we recommend that at least 16 ELNs be removed for accurate nodal staging as well as for obtaining a therapeutic benefit after nCRT for EC. Furthermore, once precise nodal staging has been achieved, patient survival does not improve with additional ELN dissection after nCRT, regardless of pathological nodal staging (negative or positive).

## Introduction

Esophageal cancer is ranked seventh in cancer incidence rates (572,000 cases) and sixth for cancer deaths (508,000 cases) worldwide, with a poor prognosis and high mortality rate (1). For patients with potentially curable localized tumors, surgical resection via esophagectomy with two- or three-field lymphadenectomy is still the primary form of treatment, with a 5-year survival rate of 23–41% (2). With regard to the count of the examined lymph nodes, the current National Comprehensive Cancer Network (NCCN) guidelines recommend that at least 15 examined lymph nodes (ELNs) should be achieved in patients undergoing esophagectomy without preoperative treatment for adequate nodal staging (3).

For patients with locally advanced disease, neoadjuvant chemotherapy or chemoradiotherapy prior to surgery has been revealed to be associated with a significant improvement in overall survival (4, 5). Concerning the extent of lymphadenectomy, several previous studies have demonstrated that high lymph node retrieval was associated with accurate staging and excellent outcomes (6–9). Kamel MK and colleagues (10) analyzed the National Cancer Database of 14,503 esophageal cancer patients and revealed that the cutoff number of resected LNs after induction chemoradiation that was associated with the highest survival benefit was 20 nodes. In contrast, another study conducted by Zhan et al. (11) demonstrated that 18, 19, and 28 ELNs could achieve accurate N0, N1, and N2 staging for patients with neoadjuvant therapy. However, the optimal count of ELN dissection post nCRT for carcinoma of the esophagus has not yet been well established. Moreover, the real value of extended lymphadenectomy following neoadjuvant treatment in EC is still elusive and debatable.

The purpose of the present study was to use the Surveillance, Epidemiology, and End Results (SEER) database to investigate the optimal count of ELN dissection and the real value of extended lymphadenectomy among patients who underwent neoadjuvant treatment followed by esophagectomy with lymphadenectomy at a population-based level.

## Materials and Methods

### Patient Population

All cases were obtained from the National Cancer Institute’s SEER database (http://www.seer.cancer.gov) with the use of SEER*Stat version 8.3.8 software: SEER 18 Regs Custom Data (with additional treatment field), Nov 2018 Sub (1975–2016 varying) database. Using the “Primary Site-labeled” variable, we chose tumor cases from the primary site of the esophagus diagnosed between 2001 and 2016. Only patients who received radiation (with or without chemotherapy) prior to surgery were included in this study. Patients with any of the following criteria were excluded: surgery alone, unknown regional nodes examined or positive, and unknown survival time. Information on the age at diagnosis, sex, race, size of the tumor, histologic type, grade, primary site, American Joint Committee on Cancer (AJCC) stage (6th ed or 7th ed), radiation sequence with surgery, chemotherapy recode (yes or no), regional nodes examined, regional nodes positive, distant metastasis, cause of death, and survival time was collected. Patients were staged according to the seventh edition of the TNM classification.

Approval for the study by the local institutional review board and written consent were not required because it was a public database of clinical research.

### Statistical Analysis

Categorical data were compared using Pearson chi-square tests or Fisher’s exact test, and continuous data were compared using ANOVA. A binary logistic regression model and Cox proportional hazards regression were carried out to determine the correlation between ELNs and stage migration (node negative versus node positive) and OS. The survival rate was calculated using the Kaplan-Meier method, and a log-rank test was used to assess the survival differences between groups. Statistical analyses were performed by using IBM SPSS 25.0 software (IBM, Inc.). *P* values of less than 0.05 were considered statistically significant.

In accordance with the procedures previously described by Liang et al. (12), the curves of the odds ratios (ORs; stage migration) of each ELN count compared with one ELN (as a reference) were smoothed using the locally weighted scatter-plot smoother (LOWESS) technique with a bandwidth of 2/3 (default). The structural break points were determined by the Chow test, which were considered the threshold of clinical impact. Then, the curves of hazard ratios (HRs; OS) of more ELN counts compared with the threshold ELN count (as a reference) were smoothed to characterize the relationship between survival and the extended ELN yield. Both the LOWESS and Chow tests were performed by using Python version 3.7 software (Python Software Foundation, Delaware).

## Results

### Patient Characteristics

**Figure 1:**
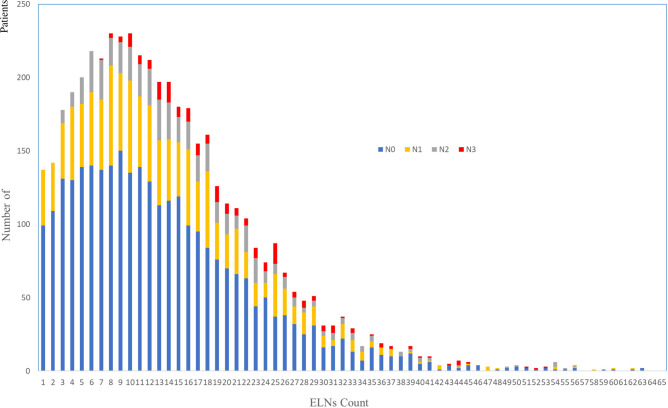
Distribution of examined lymph nodes.

A query of the SEER Database resulted in a total of 4,710 patients who received nCRT followed by esophagectomy that were performed for cancer between 2001 and 2016. The characteristics of the patients are described in **Table 1**. The study group consisted of 750 women and 3,960 men, with a median age of 61 years (range: 23–88) and a distinct preponderance of adenocarcinoma histology (adenocarcinoma: others, 3.5:1). The distribution of ELNs is shown in **Figure 1**, with a median number of harvested ELNs of 12 (range: 7–19). Furthermore, the median number of ELNs increased from 9 in 2001 to 2004 to 15 in 2013 to 2016.

**Table 1:** Patient demographics.

Characteristic	Value
Number of patients	4,710
Age (years)	
Median (range)	61 (23–88)
Sex	
Male	3,960
Female	750
Race	
White	4,292
Black	233
Others	185
Tumor location	
Upper third	76
Middle third	534
Lower third	3,824
Esophagus, NOS	224
Histology	
AC	3,664
SCC	943
Others^a^	63
Carcinoma, NOS	40
Tumor cell differentiation	
Well differentiated	223
Moderately differentiated	1,980
Poorly differentiated	2,507
T stage	
T0	2
T1	471
T2	628
T3	2,343
T4	235
Unknown	1,031
N stage	
N0	2,918
N1	1,124
N2	480
N3	188
Median HLN count (IQR)	
Overall	12 (7–19)
No. regroup (2001–2004)	9 (5–14)
No. regroup (2005–2008)	10 (6–17)
No. regroup (2009–2012)	13 (7–20)
No. regroup (2013–2016)	15 (10–21)
Year of diagnosis	
2001–2004	718
2005–2008	1,027
2009–2012	1,288
2013–2016	1,677

*AC, Adenocarcinoma; SCC, Squamous Cell Carcinoma; NOS, not otherwise specified.*

^a^ *Includes neuroendocrine carcinoma, small cell carcinoma, large cell neuroendocrine carcinoma.*

### Correlation between ELNs and Stage Migration

The median number of ELNs differed significantly within subgroups of the histologic type (AC: SCC: others: NOS, 14.3:13.2:13.9:10.7; *P *= 0.01), T stage (T0: T1: T2: T3: T4: TX, 10.0:13.1:13.8:14.8:13.7:13.3; *P *< 0.01) and N stage (N0: N1: N2: N3, 13.4:13.9:16.0:21.7; *P *< 0.01). There was a significantly proportional increase in the N stage (from N0 to N1, N2, and N3), with an increasing ELN count after adjusting for the T stage (OR, 1.017; 95% CI, 1.011 to 1.023; *P *< 0.001; **Table 2**). However, after stratification by histologic type, only a consistent trend was observed in patients with AC with an increasing ELN count (OR, 1.021; 95% CI, 1.014 to 1.028; *P *< 0.001).

**Table 2:** Number of ELNs (as a continuous variable) and Stage Migration and OS, Stratified by Histologic Subgroups, Adjusted for T stage.

Histology	Stage Migration		OS (Node-Negative)		OS (Node-Positive)	
	Sig.	OR (95% CI)	Sig.	HR (95% CI)	Sig.	HR (95% CI)
Overall	<0.001	1.017 (1.011–1.023)	<0.001	0.983 (0.977–0.988)	<0.001	0.986 (0.981–0.992)
AC	<0.001	1.021 (1.014–1.028)	<0.001	0.981 (0.974–0.988)	<0.001	0.985 (0.979–0.992)
SCC	0.649	1.003 (0.989–1.018)	0.011	0.986 (0.975–0.997)	0.925	0.999 (0.984–1.015)
Other^a^	0.926	0.997 (0.945–1.052)	0.773	0.993 (0.944–1.044)	0.056	0.941 (0.884–1.002)

*AC, Adenocarcinoma; CI, Confidence Interval; HR, Hazard Ratio; OS, Overall Survival; OR, Odds Ratio; SCC, Squamous Cell Carcinoma.*

^a^ *Includes neuroendocrine carcinoma, small cell carcinoma, large cell neuroendocrine carcinoma and NOS.*

### Correlation between ELNs and OS

After adjusting for the T stage, a greater number of ELNs was significantly associated with a better OS among patients with node-negative (N0) disease (HR, 0.983; 95% CI, 0.977 to 0.988; *P *< 0.001) and patients with node-positive (N1, N2, and N3) disease (HR, 0.985; 95% CI, 0.979 to 0.992; *P *< 0.001). However, after stratification by histologic type, only a consistent trend was observed in patients with AC, with the exception of node-negative patients with SCC (HR, 0.986; 95% CI, 0.975 to 0.997; *P *< 0.001; **Table 2**).

### Cut-Point Analysis for Nodal Stage Migration and Validation

**Figure 2:**
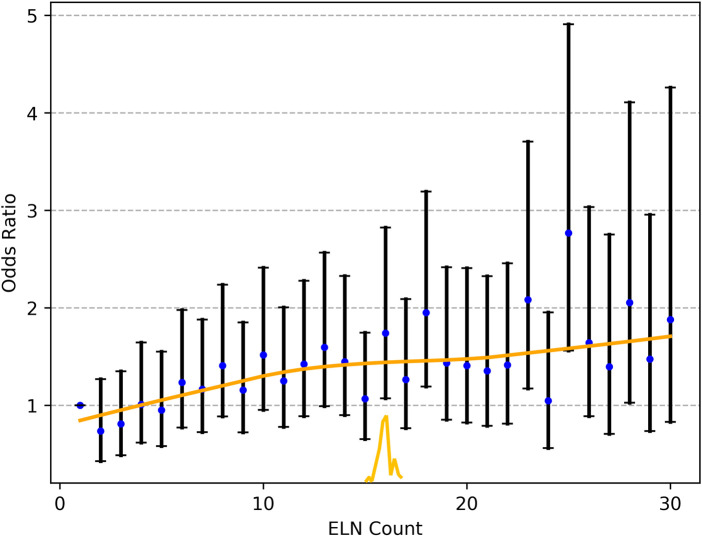
The fitting curves for the OR of nodal stage migration were smoothed using the LOWESS technique, and the corresponding cutoff point of the ELNs was calculated by the Chow test. ELN, examined lymph node. OR, odds ratio.

As shown in **Figure 2**, the fitting curves for the OR of nodal stage migration were smoothed using the LOWESS technique. The corresponding cutoff point of 16 ELNs was calculated for the OR of stage migration by the Chow test.

**Figure 3:**
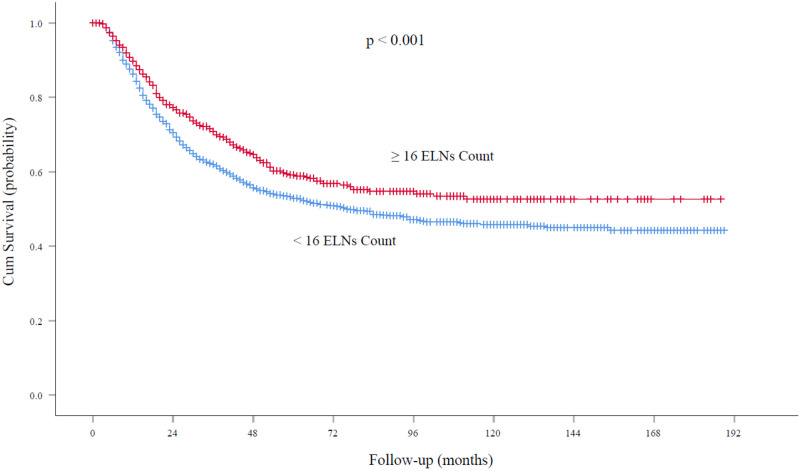
Overall survival rates among patients with node-negative EC at the cutoff point of 16 ELNs. ELN, examined lymph node. EC, esophageal cancer.

**Figure 4:**
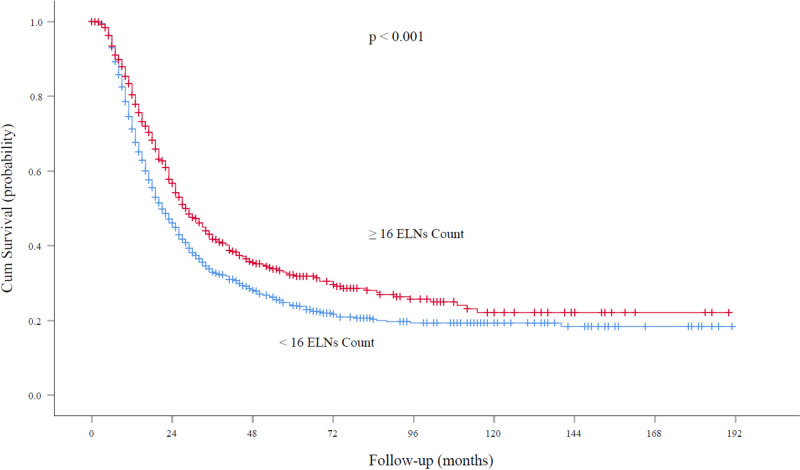
Overall survival rates among patients with node-positive EC at the cutoff point of 16 ELNs. ELN, examined lymph node. EC, esophageal cancer.

At the time of analysis, the median follow-up was 62.0 months. For node-negative patients, the five-year survival rates and median survival time were 59.2% and 72.0 months (95% confidence interval, 61.5 to 79.5 months), respectively, among patients with ELN count ≥16 and 52.9% and 48.6 months (95% confidence interval, 40.0 to 52.5 months), respectively, among those with ELN count <16 (*P* < 0.001; **Figure 3**). With regard to node-positive patients, the five-year survival rates and median survival time were 32.2% and 29.0 months (95% confidence interval, 25.7 to 32.2 months), respectively, among patients with ELN count ≥16 and 24.2% and 21.0 months (95% confidence interval, 18.8 to 23.1 months), respectively, among those with ELN count < 16 (*P* < 0.001; **Figure 4**).

### Impact of Extended Lymphadenectomy

**Figure 5:**
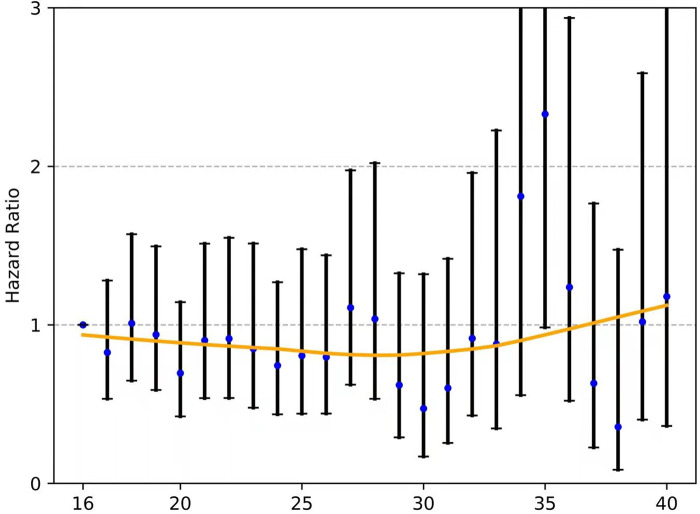
The fitting curves for the HR of each ELN count (>16 nodes) compared with 16 ELNs (as a reference) among patients with node-negative EC were smoothed using the LOWESS technique. ELN, examined lymph node. EC, esophageal cancer. OR, odds ratio.

**Figure 6:**
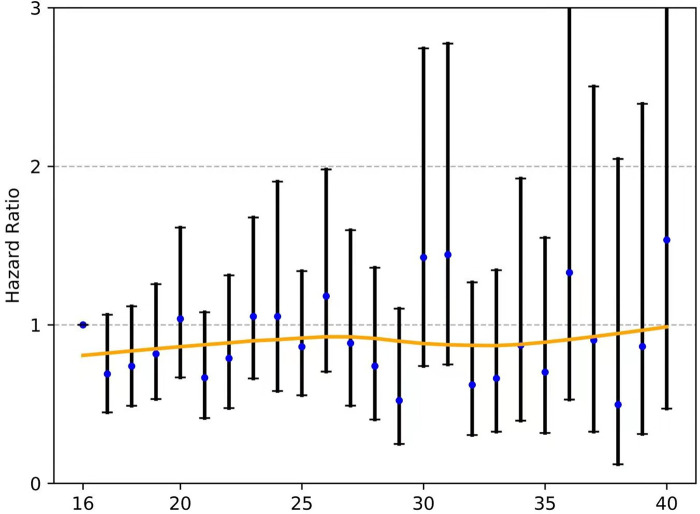
The fitting curves for the HR of each ELN count (>16 nodes) compared with 16 ELNs (as a reference) among patients with node-positive EC were smoothed using the LOWESS technique. ELN, examined lymph node. EC, esophageal cancer. OR, odds ratio.

For those with node-negative and node-positive diseases, the curves of the hazard ratios (HRs; OS) of each ELN count (>16 nodes) compared with 16 ELNs (as a reference) were smoothed using the LOWESS technique and are shown in **Figure** 5 and Figure 6, respectively; however, they failed to demonstrate a significant trend of survival benefit that favored a more extensive lymphadenectomy (HR, 1.001; 95% CI, 0.989 to 1.012; *P *= 0.906; and HR, 0.996; 95% CI, 0.985 to 1.006; *P *= 0.405, respectively).

## Discussion

The importance of the number of ELNs during esophagectomy alone for staging accuracy and long-term survival has been well documented and acknowledged (13–18). Accordingly, the NCCN guidelines recommend that at least 15 lymph nodes (LNs) should be removed in patients undergoing esophagectomy without preoperative treatment for adequate nodal staging (3). However, the impact of neoadjuvant treatment on the dissemination and the pattern of lymph node distribution has not been well established. Moreover, the potential effect of extensive lymphadenectomy on survival after surgery following neoadjuvant therapy remains controversial. The optimum lymphadenectomy during esophagectomy in patients after receiving neoadjuvant treatment has not yet been well identified. The study by Castoro and associates (19) on 402 patients with cancer of the esophagus or esophagogastric junction demonstrated that not only did the frequency of lymph node metastases decrease after neoadjuvant chemoradiotherapy (nCRT) but also the nodal localization and pattern were significantly modified. Moreover, in the Dutch CROSS trial, the median (interquartile range) number of resected nodes for patients who underwent surgery alone and received multimodality treatment was 18 (12–27) and 14 (9–21), with 2 (1–6) and 0 (0–1) resected positive nodes, respectively (20). Similarly, randomized clinical trials published in the New England Journal of Medicine have also revealed that there was a significant trend toward less advanced nodal disease in the perioperative chemotherapy group than in the surgery group (84.4% vs. 70.5%, *P *= 0.01) (4). Therefore, the question arises whether suboptimal lymphadenectomy affects the accuracy of nodal stage or long-term outcome after nCRT in patients with EC.

A limited number of LNs harvested in EC patients after nCRT may lead to inaccurate nodal staging, particularly with regard to those with nodal upstaging. As Meguid et al. (21) reported, almost seventy percent of EC patients are partial responders and nonresponders to nCRT. In the current study, the US SEER Database was analyzed and the data clearly indicated that a larger number of LNs dissection was correlated with a higher proportion of more advanced nodal stage cases in SCC and AC post neoadjuvant treatment. Furthermore, our results suggested that at least 16 nodes should be removed to avoid nodal staging migration during esophagectomy for patients undergoing nCRT, which is mainly consistent with the NCCN guideline recommendations for those undergoing surgery alone (15 ELNs). Similarly, Stiles et al. (7) revealed that optimal lymphadenectomy, as defined by the WECC, may also be applicable to cases of esophagectomy following neoadjuvant therapy, particularly those who were not downstaged by pathological tumor depth classification and those with persistent nodal metastases.

A second question concerning long-term effects is whether overall survival may be improved by extended lymphadenectomy for patients treated with nCRT. Some investigators believe that resecting more LNs is helpful for accurate nodal staging and decreasing the chance of undiscovered positive LNs. For patients with node-positive disease (not declared as node-negative disease due to fewer ELNs), adjuvant treatment after surgery could improve the OS. In addition, extended nodal resection would benefit the clearance of micrometastases and residual lesions after neoadjuvant therapy and reduce the risk of local recurrence. For example, Solomon and colleagues (22) analyzed the SEER database of 4,224 patients and showed that there is a cooperative survival benefit for neoadjuvant radiation and adequate lymphadenectomy (ELNs ≥18) in patients with node-positive esophageal adenocarcinoma. Furthermore, a meta-analysis conducted by Visser et al. (8) demonstrated a survival benefit of high lymph node yield on overall survival (HR = 0.82; 95% CI = 0.73–0.92; *P* < 0.01). In contrast, however, in the present study, our results showed that once precise nodal staging (>16 nodes) had been achieved, patient survival did not improve with additional LNs dissection after nCRT, regardless of nodal stage (negative or positive). As stated by, the total number of ELNs was significantly associated with survival for patients in the surgery-alone arm (*P* = 0.007) but not in the nCRT arm (*P* = 0.98). Similarly, Shridhar et al. (23) noted that the number of LNs harvested during esophagectomy after nCRT does not impact OS or disease-free survival (DFS). Furthermore, Noordman BJ et al. (24) revealed that compared to surgery alone, the addition of nCRT may reduce the need for extended lymphadenectomy to improve long-term survival in patients with esophageal adenocarcinoma. This difference in effect of lymphadenectomy might be partially explained by the sterilization of micrometastases after nCRT and high local tumor control rate of radiotherapy on the regional lymph nodes. It has been reported that the pathological complete response rate was 19–40% in neoadjuvant CRT (25, 26). Moreover, several previous studies (24, 25) revealed that the number of resected LNs and the number of resected positive LNs were significantly decreased in patients treated with nCRT than in those treated with surgery alone, suggesting that the clinical importance of extended lymphadenectomy to improve the locoregional control rate may differ between surgery after nCRT and surgery alone. In addition, As Patti et al. (27) demonstrated, it is not the extent of lymphadenectomy that dictates outcomes but rather the tumor biology and the stage of the tumor during the surgery. In addition, a more extensive lymphadenectomy does have the disadvantage of greater surgical morbidity, which confers a large negative impact on survival after esophagectomy for cancer (8, 28). Further studies with larger study populations or other methods are required to better elucidate the role of extended lymphadenectomy following neoadjuvant treatment.

Finally, it should be noted that there are several other factors that may influence the long-term outcomes in patients undergoing lymphadenectomy post nCRT, including ELN location and ratio and extracapsular ELN involvement. In detail, a study by Phillips (6) concluded that omitting lymphatic dissection of hepatic, celiac, and splenic nodes may lead to a very limited number of extra cancer-related deaths, and thus, the extent of lymphadenectomy post neoadjuvant chemotherapy must be correlated with the node location. With regard to the ELN ratio, Mariette and colleagues (29) demonstrated that the ratio between metastatic and examined lymph nodes is an important predictor of a poor prognosis, especially for inadequately staged EC patients following nCRT. In addition, D’Journo et al. (30) found that extension of tumor cells through the nodal capsule into the fatty tissue seems to be an independent negative prognostic factor affecting survival in patients with locally advanced EC treated with nCRT and surgery.

This study has several limitations. First, it was retrospective, and some important patient clinicopathological features were rather limited, such as the variations in radiation doses and range over the study period, the usage of different combinations of chemotherapeutic agents, various therapeutic modalities given following surgery, various degrees of pathologic tumor response to neoadjuvant therapy, the incidence of postoperative complications, the lack of standardized staging due to missing data of clinical TNM stage, and resection margin status (R0, R1 or R2), all of which have the potential to modulate the treatment effect. Additionally, owing to the limitations of the database, we did not further associate the number of ELNs with their locations (cervical, mediastinal or abdominal), surgical approach (transthoracic or transhiatal), or surgeon/hospital esophagectomy volume, which might affect the ELN status and long-term outcomes as previously reported. We suggest a future prospective randomized trial with a larger study population or other methods to validate our results.

In conclusion, our data suggest that at least 16 ELNs should be achieved for accurate nodal staging as well as for obtaining a therapeutic benefit for patients undergoing nCRT. Furthermore, once precise nodal staging has been achieved, patient survival does not improve with additional LNs dissection after nCRT, regardless of the pathological nodal staging (negative or positive). These findings have important clinical implications and should be considered when performing esophagectomy for patients post nCRT.

## Data Availability

The raw data supporting the conclusions of this article will be made available by the authors, without undue reservation.
